# Speckle Tracking Echocardiography in Patients with Ankylosing Spondylitis and Evaluation of Subclinical Involvement

**DOI:** 10.31138/mjr.20230724.ei

**Published:** 2023-07-24

**Authors:** Mir Amir Aghdashi, Amir Mikaeilvand, Mohammad Mostafa Ansari-Ramandi, Ali Khodaparast, Alireza Rostamzadeh

**Affiliations:** 1 Department of Rheumatology, Faculty of Medicine, Urmia University of Medical Sciences, Urmia, Iran,; 2 Department of Cardiology, Urmia University of Medical Sciences, Urmia, Iran,; 3 Cardiovascular Diseases Research Centre, Birjand University of Medical Sciences, Birjand, Iran,; 4 Urmia University of Medical Sciences, Urmia, Iran

**Keywords:** speckle tracking, global longitudinal strain, rheumatology, aortic root, ankylosing spondylitis

## Abstract

**Introduction:**

Ankylosing spondylitis (AS) is a chronic inflammatory condition primarily affecting the spine and the joints. It also affects multiple organs in the body including the cardiovascular system. Left ventricular (LV) global longitudinal strain (GLS) is a good measure for recognizing subclinical myocardial dysfunction. This study aimed to investigate if there is sub-clinical LV myocardial systolic dysfunction present in AS patients independent of the presence of cardiovascular disease risk factors. We also aimed to see if the GLS is associated with the aortic root abnormality present in these patients.

**Methods:**

Twenty-eight AS patients (mean age 40.6±10.9 years) were investigated in this cross-sectional case-control study. The control group (mean age 45.2±5 years) comprised 26 healthy individuals. Conventional and speckle tracking echocardiography was performed for all patients. LV systolic myocardial function was assessed by systolic GLS. Aortic diameters and diastolic function were also evaluated.

**Results:**

The baseline characteristics and cardiovascular risk factors of the case and control groups were similar and did not differ significantly. The AS patients were suffering more from diastolic dysfunction in comparison to the control group (p=0.009). We only found a significantly impaired longitudinal strain in the 3-chamber view of AS patients when compared to the control group. There was no significant association between the GLS and aortic root abnormality.

**Conclusion:**

Although the impaired longitudinal strain present in AS patients is not associated with the aortic root abnormality, it can be an early sign of cardiovascular involvement.

## INTRODUCTION

Ankylosing spondylitis (AS) is a chronic inflammatory condition that affects many joints including the sacroiliac and peripheral joints and the spine.^1^ AS is also associated with extra-spinal manifestations including the involvement of different organs in the body such as the gastrointestinal tract, eyes, cardiovascular system, pulmonary system, renal system, and the brain.^1^ Rheumatic and inflammatory diseases can also cause an increased risk of CVD without having the traditional risk factors.^2^

The prominent cardiac involvement in AS patients is the inflammation in the aorta called aortitis. This condition leads to aortic root dilation and aortic valve insufficiency.3 The other cardiac manifestations of AS are left ventricular (LV) systolic and diastolic dysfunction, LV hypertrophy, atherosclerosis, and valvular diseases.^1^ For evaluating the LV systolic function some studies have used conventional echocardiography,^1,4–6^ while limited studies have used speckle tracking echocardiography (STE).^3,7^ Echocardiographic strain imaging is a method that has been developed for the evaluation of regional myocardial function. Speckle Tracking Echocardiography (STE) is a post-processing computer algorithm that uses grayscale digital images to strain analysis. The grayscale digital images of the myocardium have a unique speckle pattern and the algorithm automatically subdivides regions into units of pixels tracking stable patterns of speckles. Then the algorithm measures the absolute differences by searching for the new location of the speckle patterns within each of the units. STE is used to detect subclinical LV ventricular dysfunction before the development of its clinical form with high sensitivity and accuracy.^8^

Apart from the limited studies using STE in evaluating myocardial function in AS patients, whether subclinical left ventricular systolic dysfunction is present in AS patients in our population independent of the presence of CVD risk factors is not known. In this study, we aimed to address this issue and see if the subclinical LV myocardial dysfunction is associated with the aortic root abnormalities in these patients.

## MATERIALS AND METHODS

### Study population

In this case-control study, conducted from March 2017 to March 2018, we recruited 28 patients with AS and 26 healthy individuals as controls. All cases with AS were consecutively selected from among the patients who visited the clinic of the rheumatologic disease in a tertiary care centre. The diagnosis of AS was based on the modified New York criteria for AS.^9^

### Inclusion and exclusion criteria

Inclusion criteria were patients between the age of 18 and 65 who were referred for cardiovascular evaluation by the rheumatologist. Exclusion criteria consisted of diabetes mellitus, hypertension, chronic pulmonary diseases, dyslipidaemia, chronic kidney disease, and cardiovascular diseases (CVDs) or the consumption of cardiovascular medications. Asymptomatic individuals without a history of CVDs or risk factors were selected as controls.

### Paraclinical evaluation

All the subjects were examined using echocardiography and electrocardiography (ECG) for an evaluation of their cardiac status.

### Echocardiography

The echocardiography was done by a cardiologist with echocardiography fellowship using a GE Vivid 7 and assessments were done for cardiac function, valvular involvement, aortic annulus size, sinus of Valsalva size, and aortic root size and diastolic function, and speckle tracking. The ejection fraction (EF) of the LV was measured using the Simpson method and the value bigger than or equal to 55% was marked as a normal ejection fraction. The valvular evaluation was performed by colour and doppler study and valvular stenosis or regurgitation more than mild was marked significant valvular disease. All images were taken in 3 separate beats and then the measurements were averaged. For speckle tracking the frame rate was 50 to 90 frames per second and the endocardial line was defined manually. Peak longitudinal strain was evaluated in two, three, and four-chamber views, and then the global longitudinal strain was derived. Diastolic function was evaluated by mitral valve flow E-wave velocity ratio to A-wave velocity and a number less than 0.8 was regarded as grade I diastolic dysfunction; the number equal to or above 0.8 was marked as no diastolic function or pseudo-normal diastolic function.

### ECG

Standard 12-lead ECGs were obtained using a recorder at a paper speed of 25 mm/s and a scale of 10 mm/mV standardisation. The interpreter was a cardiologist blinded to the clinical status of the patients.

### Statistical analysis

The categorical data were presented as numbers (percentages) and the continuous variables as the mean ± standard deviation or the median (interquartile ranges 25–75%), as appropriate. The continuous variables were compared between the groups using the t-test or the Mann–Whitney U tests, as appropriate. The χ^2^ test or the Fisher exact test were used for analysing the categorical variables. The data were analysed with the Statistical Package for the Social Science Base, version 21.0 (SPSS, IBM, NY). The level of significance was 0.05.

## RESULTS

There were 26 persons (18 (69.2%) men) in the control group and 28 cases (22 (78.6%) men) in the AS group. The age of the individuals was 45.27±5.06 years in the control group in comparison to 40.64±10.94 years in the AS group. All demographic data including height and weight was not significantly different across the groups. (**Table 1**)

**Table 1.** Demographic data.

		**Mean**	**Standard deviation**	**Minimum**	**Maximum**
Age	Control group	45.27	5.06	38	55
	AS group	40.64	10.94	21	63
Height	Control group	171	4.69	165	180
	AS group	168.35	6.82	150	178
Weight	Control group	73.69	5.61	65	85
	AS group	72.5	9.95	50	89

All individuals in control and 26 persons (92.8%) in AS (Ankylosing Spondylitis) group had a normal systolic left ventricular function. (P-value: 0.033). The sinus of Valsalva diameter was significantly (p value= 0.024) increased in the AS patients in comparison to in the control group with a value of 3.86±0.22 cm and 2.64±0.37 cm respectively. Aortic root size and aortic annulus size were not significantly different (P-value: 0.32 and P-value: 0.959, respectively). There was no significant valvular disease in both groups.

The 3-chamber longitudinal strain was significantly (p value=0.035) impaired in the AS group in comparison to the control group with a value of −17.11±7.17 and −20.55±1.57 respectively. The 2-chamber longitudinal strain (P-value: 0.565), 4 chamber longitudinal strain (P-value: 0.772) and average longitudinal strain (P-value: 0.227) were not significantly different.

Grade I diastolic dysfunction (E/A< 0.8) was significantly more in the AS group with 20 (71.4%) AS patients having grade I diastolic dysfunction in comparison to 8 (31%) in the control group (p value=0.009). Grade II and III diastolic dysfunction were not evaluated and E/A> 0.8 was marked as no diastolic dysfunction or pseudo-normal findings. The echocardiographic data of the groups are shown in **Table 2**.

**Table 2.** Echocardiographic findings.

	**Case (Ankylosing Spondylitis) group**	**Control group**	**P-value**
Normal LV ejection fraction (≥ 55%) (Number –[percent])	26 (92.8)	26 (100)	0.033
Aortic valve annulus diameter (cm) (mean± SD)	2.12±0.22	2±0.12	0.959
Sinus of Valsalva diameter (cm) (mean± SD)	3.86±0.22	2.64±0.37	0.024
Aortic root diameter (cm) (mean± SD)	3±0.41	3±0.31	0.320
2 chamber longitudinal strain (mean± SD)	−19.7±6.15	−20.63±1.75	0.565
3 chamber longitudinal strain (mean± SD)	−17.11±7.17	−20.55±1.57	0.035
4 chamber longitudinal strain (mean± SD)	−22.54±4.07	−20.01±1.83	0.772
Global longitudinal strain (mean± SD)	−21.3±4.57	−20.59±1.18	0.227

ECG data are listed in **Table 3**. There was no significant difference in ECG findings between the two groups.

**Table 3.** ECG findings.

**ECG findings**	**Ankylosing Spondylitis group**	**Control group**	**P-Value**
Heart Rate (mean [SD])	76.07 (12.33)	73.82 (11.25)	0.46
PR duration (mean [SD])	155.92 (25.09)	150.28 (28.38)	0.39
QRS duration (mean [SD])	80.92 (17.75)	75.29 (16.62)	0.29
QTc duration	406.46 (19.57)	404.70 (11.78)	0.14
T changes (Number [percent])	1 (3.5%)	0	0.52
ST change (Number [percent])	0	0	1

## DISCUSSION

The most common types of cardiac involvement in patients with AS are aortic root dilation, aortic valve insufficiency, and conduction abnormalities.^1^ In this study, AS patients had increased sinus of Valsalva diameter but the difference in other aortic sizes was not significant. In a study done in Norway by Helga Midtbo and colleagues, lower global longitudinal strain was associated with larger aortic root diameters.^7^

Regarding left ventricular function, we found an impaired 3-chamber longitudinal strain in AS patients in comparison to the control group. In a similar study done by Sadık Volkan Emren and his colleagues,^10^ it was shown that global longitudinal strain is impaired in AS patients, and they proposed it as a tool to differentiate between radiographic axial spondyloarthritis (AS) and non-radiographic axial spondyloarthritis. They also showed that even in the absence of cardiovascular risk factors or disease there is subclinical myocardial dysfunction in these patients.^10^ In another study by Yan Chen and his colleagues, speckle tracking echocardiography has been proposed as a tool for early detection of myocardial dysfunction in such patients.^11^ Another research team in China concluded that longitudinal strain impairment can be used as an independent predictor for future major adverse cardiovascular events in AS patients.^12^ Selin Ozen and colleagues also demonstrated that increased left ventricular global longitudinal strain is seen in AS patients.^13^ Apart from subclinical myocardial dysfunction many studies have also showed subclinical atherosclerosis in AS patients.^11–15^

However, our study showed that the impaired longitudinal strain did not have any association with the aortic root abnormalities we see in these patients.

Diastolic dysfunction was also shown to be more in the AS patients in comparison to the control group in our study which is similar to the results of the Almasi S and colleague’s study.^1^ Moyssakis and colleagues found that markers of cardiac relaxation impairment were significantly increased in AS patients in comparison to the control group and it was similar to our study results about left ventricular diastolic dysfunction.^16^ Also another study found that left ventricular diastolic function is impaired in AS patients.^17^

The exact reason for myocardial dysfunction in AS patients is unknown, but referring to some studies on the inflammatory disease, it seems systemic inflammation may implicate myocardial function,^18^ although in Emren SV and colleagues’ study an exact relationship between markers of inflammation and markers of left ventricular dysfunction was not found.^10^

## LIMITATIONS

The limitations of our study include its single-centre nature and the limited number of AS patients present. A multicentre study recruiting a larger number of patients can help in identifying any possible associations between the subclinical myocardial dysfunction in AS patients and future events in these patients.

## CONCLUSION

Despite confirmed aortic inflammation in Ankylosing Spondylitis, there was no association found between impaired longitudinal strain and increased aortic root diameter. Although the subclinical systolic myocardial dysfunction cannot be neglected in AS patients. Concerns should rise about both the LV diastolic and the systolic dysfunction present in these patients and their early detection to avoid cardiovascular consequences.

## DISCLAIMERS

The datasets generated during the current study are available from the corresponding author on reasonable request.

All authors read and approved the final manuscript.

The authors have no relevant financial or non-financial interests to disclose.

The authors declare that no funds, grants, or other support were received during the preparation of this manuscript.

This study approval was granted by the Ethics Committee of Urmia University of Medical Science.

Informed consent was obtained from all individual participants included in the study.
